# Blood flow restriction training enhances punching force and upper body strength in elite boxers: a randomized trial

**DOI:** 10.3389/fphys.2025.1693271

**Published:** 2025-10-17

**Authors:** Gaurav Awana, Moattar Raza Rizvi, Ankita Sharma, Mohammed Aldalaykeh, Zoya Zaidi, Simran Makhija, Waqas Sami, Noof Fahad A. Al-Kuwari

**Affiliations:** ^1^ Department of Physiotherapy, School of Allied Health Sciences, Manav Rachna International Institute of Research and Studies (MRIIRS), Faridabad, India; ^2^ Allied Health Sciences, Santosh Deemed to be University, Ghaziabad, Delhi, India; ^3^ Department of Physiotherapy, Amity Institute of Health Allied Sciences, Amity University Uttar Pradesh, Noida, India; ^4^ College of Nursing, QU-Health Sector, Qatar University, Doha, Qatar; ^5^ Physiocentric Clinic for Sports Injury Management, Manual and Exercise Therapy, Gulmohar Park, New Delhi, India; ^6^ Department of Pre-Clinical Affairs, College of Nursing, QU-Health Sector, Qatar University, Dohai, Qatar

**Keywords:** blood flow restriction training, punch force, upper limb strength, combat sports, boxing, resistance training

## Abstract

**Introduction:**

Boxing demands explosive punching force, yet heavy resistance training risks joint stress and fatigue. Blood Flow Restriction Training (BFRT) offers a low-load alternative that stimulates strength and power gains. This randomized controlled trial investigated BFRT’s effects on upper limb strength and punching force in elite amateur boxers, aiming to establish its value as a safe, performance-enhancing strategy.

**Methods:**

Thirty elite male amateur boxers (≥3 years of competitive experience) were randomized into an experimental group (BFRT) or a control group. Both groups completed identical upper-body resistance exercises thrice weekly for 8 weeks, The control group trained at 50%–60% 1RM (one repetition maximum), while the BFRT group trained at 20%–30% 1 RM with 40%–50% limb occlusion pressure using standardized 7 cm cuffs. Primary outcomes included 1RM, strength (elbow flexion, extension, bench press) and peak punch force (jab, cross, uppercut, hook) measured via a calibrated vertically-mounted force plate.

**Results:**

After 8 weeks, the BFRT group demonstrated significantly greater gains in dominant-arm strength, with 1RM elbow flexion increasing by +3.3 kg (p < 0.001, d = 3.20), elbow extension by +2.95 kg (p < 0.001, d = 2.84), bench press by +13.6 kg (p < 0.001, d = 1.81), and squat by +15.6 kg (p < 0.001, d = 2.05) compared with smaller improvements in controls. Peak Punch force improved markedly in the BFRT group: jab +895 N (p = 0.001, d = 1.52), uppercut +1142 N (p < 0.001, d = 3.02), hook +1157 N (p < 0.001, d = 2.55), and cross +1067 N (p < 0.001, d = 3.80). Repeated-measures ANOVA confirmed strong group × time interaction effects (η^2^ = 0.27–0.87).

**Conclusion:**

BFRT led to substantial improvements in upper limb strength and peak punching force in elite boxers using a low-load protocol. These findings suggest BFRT is a safe, effective training strategy that may enhance sport-specific power outputs while potentially reducing joint stress, making it a valuable addition to high-performance boxing programs.

## 1 Introduction

Boxing is a dynamic combat sport that requires athletes to generate powerful and precise punches through the coordinated activation of their lower limbs, core, and upper extremities. The technical execution of a punch originates from the ground, is transmitted through the kinetic chain, and culminates in the arm’s rapid movement (Dinu and Louis, 2020; Lenetsky et al., 2013). According to Newton’s second law, the acceleration of a punch is proportional to the force applied, underscoring the importance of both muscle strength and movement velocity in maximizing punch effectiveness (Dunn et al., 2022; Stanley, 2020). Optimizing punching force is not only essential for scoring, but also for ensuring consistent performance across rounds in elite boxing competitions.

Numerous training modalities such as high-load strength training, ballistic movements, plyometrics, and Olympic lifting have been widely used to enhance strength and power in athletes, including boxers (Kruszewski et al., 2016; Loturco et al., 2016; Yi et al., 2022; Yi et al., 2023). While these approaches increase maximal and explosive strength—both crucial for punch output—they also introduce cumulative stress on joints and soft tissues, thereby raising the risk of injury. In high-performance settings, this can interfere with technical training, increase fatigue, and limit training adherence. Therefore, developing alternative training strategies that improve strength while minimizing mechanical load is a priority in boxing conditioning.

Blood Flow Restriction Training (BFRT) has emerged as a promising modality capable of enhancing muscular strength and hypertrophy using loads as low as 20%–30% of one-repetition maximum (Bowman et al., 2019; Pignanelli et al., 2021; Yasuda et al., 2015). By applying pneumatic cuffs to the limbs during low-load resistance exercises, BFRT induces localized hypoxia and mechanical tension, stimulating muscular adaptations typically seen with high-intensity training. This technique has demonstrated benefits in clinical, rehabilitative, and athletic populations (Lawler et al., 1993; Lowery et al., 2014). In boxing, BFRT is particularly valuable during deloading or recovery phases, offering the opportunity to maintain or enhance muscle function without the mechanical burden of traditional training. These effects are supported by syntheses showing low-load BFRT can enhance hypertrophy, maximal strength, and, in some cases, power, via metabolic stress and fast-twitch recruitment (Hughes et al., 2017; Jessee et al., 2018; Scott et al., 2015).

Despite its growing popularity, few studies have evaluated the use of BFRT for enhancing performance in combat sports. Specifically, there is a paucity of randomized controlled trials evaluating the direct effects of BFRT on punch-specific outcomes. Prior investigations have explored the relationship between strength and punching force, yet few have tested whether low-load BFRT protocols can translate to measurable improvements in sport-specific outputs like jab, cross, hook, and uppercut forces (Kim et al., 2018; Lenetsky et al., 2020; Monfared, 2021). This lack of targeted evidence limits the adoption of BFRT in boxing-specific conditioning.

Given the importance of upper limb strength in effective punching, injury prevention, and training sustainability, establishing evidence-based protocols such as BFRT is essential. The current study addresses this gap by investigating the impact of a structured upper body BFRT protocol on upper limb strength and punch force in elite amateur boxers using a calibrated force plate. This study aimed to assess the effects of an 8-week upper extremity BFRT program on one-repetition maximum strength and punch force in elite male boxers. It was hypothesized that the BFRT group would demonstrate significantly greater improvements in both strength and punch-specific force compared to a control group undergoing similar training without occlusion.

## 2 Materials and methods

This randomized controlled trial was reported in accordance with the CONSORT 2010 guidelines for parallel-group trials, ensuring methodological transparency, completeness, and replicability of the findings.

### 2.1 Study design

**FIGURE 1:**
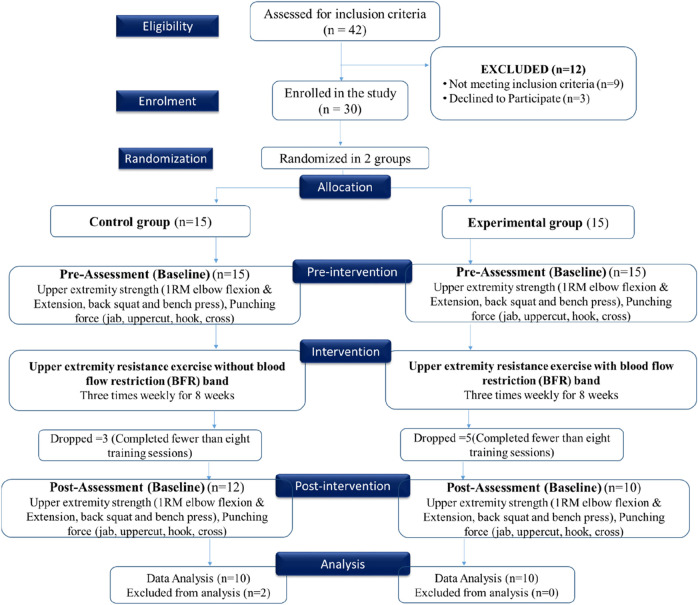
CONSORT-style flow diagram illustrating participant progression through the trial, including enrollment, randomization, group allocation, follow-up, and final analysis.

This single-blind, parallel-group randomized controlled trial evaluated the effects of Blood Flow Restriction Training (BFRT) on upper-body strength and punching force in elite amateur boxers over an 8-week intervention period. Thirty male athletes were randomly assigned (1:1) using a computer-generated block randomization sequence into either an experimental group (BFRT) or a control group (non-BFRT), with 15 participants per group (see Figure 1). Randomization was performed by an independent researcher not involved in recruitment or assessment. The study employed a single-blind design, in which the outcome assessors were blinded to the group allocation to reduce detection bias. Both groups underwent a supervised resistance training protocol three times per week with identical exercises, frequency, and supervision. The control group trained at 50%–60% of 1-repetition maximum (1RM) while the BFRT group trained at 20%–30% of 1RM under occlusion. The BFRT group performed upper body exercises under occlusion using 7 cm wide pneumatic cuffs (The Occlusion Cuff PRO, Belfast, United Kingdom) applied to the proximal arm at 40%–50% of arterial occlusion pressure. The control group completed the same exercises without occlusion. All sessions were supervised by certified strength coaches.

### 2.2 Sample size and statistical power

*A priori* sample size estimation was conducted using G*Power version 3.1.9.4 for a repeated-measures ANOVA (within-between interaction). Assuming a medium effect size (f = 0.25), an alpha level of 0.05, a power of 0.80, and a correlation among repeated measures of 0.5, the analysis indicated that a minimum of 24 participants would be required to detect a statistically significant group × time interaction. To account for an anticipated attrition rate of 20%, the final sample size was set at 30 participants (15 per group). Of these, 22 participants completed post-intervention assessments (12 in the control group and 10 in the experimental group), and 20 participants (10 per group) were included in the final analysis, which fell slightly short of the 24 required by the *a priori* power analysis.

### 2.3 Participant

Male amateur boxers aged 18–30 years were recruited from Dronacharya Boxing Academy, Haryana, India. Eligible participants had a minimum of 3 years of structured boxing experience and had competed at the state or national level in officially sanctioned amateur boxing events, consistent with elite-level classification in combat sports (Li et al., 2018). Participants were screened to ensure they were free from cardiovascular or metabolic disorders and could commit to the complete 8-week training and testing protocol. Written informed consent was obtained from all participants.

Exclusion criteria included a history of upper limb or shoulder injury in the past year, resting blood pressure >140 mmHg, heart rate >80 bpm, or recent use of performance-enhancing drugs. Additional exclusions included any diagnosed condition that could pose a contraindication for blood flow restriction, such as venous thromboembolism, sickle cell anemia, hemophilia, or other clotting disorders.

### 2.4 Interventions

A 1-week familiarization phase preceded the intervention to ensure proper technique, protocol compliance, and safety with BFR equipment. Participants were introduced to all exercises, proper cuff positioning, and movement patterns under supervision (Yasuda et al., 2011). The control group performed conventional upper limb resistance training three times a week for 8 weeks at 50%–60% of their 1RM, in addition to their regular boxing training. Exercises included bench press, dumbbell flys, front raises, side raises, biceps curls, hammer curls, triceps extensions, and back squats. Training volume progressed from three sets of 10 repetitions to 20 repetitions per set over the 8 weeks (Karanasios et al., 2022).

The experimental group followed an identical training program with the addition of blood flow restriction. Resting blood pressure was measured before each session. Blood flow restriction was applied using 7 cm-wide pneumatic cuffs (85 × 7 cm) placed at the most proximal region of both upper arms, just distal to the axillary fold. Limb occlusion pressure (LOP) was determined using a handheld Doppler ultrasound to detect the minimal pressure at which the brachial artery pulse was occluded. Training occlusion was set at 40%–50% of each participant’s LOP, as per published safety guidelines (Loenneke et al., 2011; Pignanelli et al., 2021; Yasuda et al., 2015).

Resistance in the BFRT group was set at 20%–30% of 1RM. This load range was selected based on prior BFRT studies demonstrating that training at 20%–30% of 1RM under partial occlusion is sufficient to induce hypertrophy, maximal strength, and explosive performance gains comparable to traditional high-load training (Chang et al., 2023; Hughes et al., 2017; Pearson and Hussain, 2015; Pehzaan et al., 2023). Each exercise was performed for three sets of 6–8 repetitions, with a controlled tempo (2 s concentric, 1 s eccentric). Rest intervals were 30–40 s between sets and 2–3 min between exercises. Occlusion cuffs were inflated during each exercise and deflated immediately after the set to allow for reperfusion. Total exercise duration per session ranged from 30 to 40 min. To minimize confounding variables, all participants maintained a standardized diet of ∼2,500–3,000 kcal/day, based on individual energy expenditure. Alcohol and high-caffeine beverages were restricted during the study period. Sleep patterns were monitored using wearable devices, targeting 7–8 h of sleep per night. Training volume was monitored and adjusted weekly in consultation with boxing coaches to ensure consistency across groups. These control measures aimed to isolate the effects of BFRT and reduce external variability in physical performance. In addition, participants were monitored during every training session for potential adverse reactions (e.g., discomfort, numbness, dizziness, or unusual pain), and they were encouraged to report any symptoms immediately.

### 2.5 Outcome measures

#### 2.5.1 One repetition maximum (1RM) strength measurement

Upper and lower limb strength were evaluated using a standardized 1RM protocol for key boxing-relevant exercises, including the bench press, back squat, elbow flexion (biceps curl), and elbow extension (triceps extension). Each participant began with a general warm-up followed by 5–7 repetitions at 50% of their estimated 1RM. Subsequent sets involved reduced repetitions and progressively increased loads (60%, 80%, 95%, and 100% of the initial load). If the predicted 1RM was successfully lifted, participants proceeded with incremental increases (2.5–4.0 kg) until failure. Up to three maximal attempts with 5-min rest intervals were allowed to determine peak strength (Karanasios et al., 2022; Pavlou et al., 2023). 1RM assessments were scheduled across separate days for each major muscle group to reduce fatigue effects.

#### 2.5.2 Assessment of elbow flexion and extension

Elbow flexion and extension strength were assessed explicitly due to their critical role in delivering different punch types. Biceps curls were used to evaluate elbow flexion strength, engaging the biceps brachii, brachialis, and brachioradialis. Triceps extensions were performed to assess elbow extension strength, targeting the triceps brachii. These exercises provided isolated strength measurements essential to understanding punch mechanics in boxing (Markovic et al., 2016; Stanley, 2020).

#### 2.5.3 Assessment of squat and bench press

To capture compound strength relevant to boxing, 1RM back squat and bench press were assessed. The back squat evaluated lower body power through quadriceps, hamstrings, and gluteal activation, while also involving scapular stabilizers such as the rhomboids and trapezius (Demura et al., 2010; Salyers, 2017). The bench press tested upper body pushing strength via the pectorals, deltoids, and triceps, contributing to punching power and endurance. Although additional shoulder exercises (e.g., front raise, side raise, flys) were included in training, their 1RM values were not recorded for comparative analysis (López-Laval et al., 2020).

#### 2.5.4 Measurement of punching force in boxing

Punching force was recorded using a vertically mounted Vernier force plate (Vernier, United States of America) attached to a suspended heavy bag. Calibration was conducted using 10 kg, 20 kg, and 40 kg weights, achieving linear accuracy within ±2%. The force plate was positioned at shoulder height, using the acromion process for anatomical standardization. The distance from heel to target was fixed for each athlete to minimize variability. Four punch types (jab, cross, hook, uppercut) were assessed with the dominant hand. Standardized 10-ounce gloves and hand wraps were used. Participants performed two warm-up punches followed by three maximal efforts per technique, with the highest force recorded for analysis. All athletes were tested in a neutral squared stance, with slight stance adjustment allowed during crosses for biomechanical accuracy. Hook punches were delivered against foam-padded surfaces to reduce lateral shock and injury risk (Davis, 2012; Finlay et al., 2023).

Before testing, a standardized 10-min dynamic warm-up was completed. Testing was preceded by a familiarization session 1 week earlier to minimize inter-trial variability. Standardized verbal encouragement was provided during all trials by the same assessor. To assess reliability, a subsample of 10 participants repeated punch force testing for jabs and crosses over three separate sessions spaced 24 h apart. Each participant performed three sets of three maximal punches per technique with 10-min rest intervals. Typical error and coefficient of variation were computed to evaluate intra- and inter-session consistency. This *post hoc* reliability protocol addresses current gaps in boxing-specific force plate validation literature and supports the use of this field-based method test that Blood Flow Restriction Training (BFRT) can significantly enhance punching force, upper extremity strength, and endurance (Chaabène et al., 2015).

### 2.6 Statistical analysis

All statistical analyses were conducted using IBM SPSS Statistics for Windows, Version 25.0 (IBM Corp., Armonk, NY, United States). The normality of continuous variables was assessed using the Shapiro–Wilk test, and homogeneity of variances was confirmed via Levene’s test. The assumptions of sphericity, normality, and residual variance homogeneity required for parametric tests and repeated measures analysis were verified. Between-group differences at baseline and post-intervention were evaluated using independent samples t-tests for primary outcomes, including 1RM strength (bench press, elbow flexion, elbow extension) and punching force (jab, cross, hook, uppercut). Within-group changes from baseline to post-test were analyzed using paired samples t-tests to assess training-related improvements over time. To evaluate group-by-time interaction effects, a two-way repeated measures analysis of variance (RM-ANOVA) was employed for each outcome variable. This allowed for the assessment of differential effects of the intervention over time between the BFRT and control groups. Effect sizes for t-tests were calculated using Cohen’s d (classified as small = 0.2, medium = 0.5, and large ≥0.8), while effect sizes for RM-ANOVA were expressed as partial eta squared (η^2^p) with thresholds as small (0.01), medium (0.06), and large (≥0.14). Post hoc pairwise comparisons following significant ANOVA interactions were adjusted using the Bonferroni correction. Statistical significance was set at an alpha level of p < 0.05.

## 3 Results

Of the 30 randomized participants, 8 withdrew during the intervention (3 from control, 5 from experimental), leaving 22 who completed post-intervention testing. To balance groups, 2 control participants were excluded, and 20 participants (10 per group) were included in the final analysis. No adverse reactions or safety concerns were observed or reported in either group throughout the 8-week intervention.

### 3.1 Baseline comparison

At baseline, no statistically significant differences were observed between the experimental and control groups in demographic or performance variables, confirming initial group equivalence (see Table 1). The mean age (p = 0.55), body mass index (BMI) (p = 0.85), and years of boxing experience (p = 0.213) were comparable across groups. Similarly, upper and lower limb strength measures, including 1RM elbow flexion and extension (both dominant and non-dominant sides), back squat, and bench press, showed no notable between-group differences. The only exception was a significantly higher baseline hook punching force in the dominant hand in the experimental group (mean difference = 233.4N, p < 0.001). Other punch types—jab, cross, and uppercut—demonstrated minor differences that were not statistically significant.

**TABLE 1:** Independent t-test for assessing differences in age, BMI, strength and punching force between control and experimental groups at baseline.

Outcomes	Hand	Control	Experimental	t	p	MD	95% CI		Cohen’s d
							Lower	Upper	
Age		21.60 ± 1.90	21.10 ± 1.73	0.62	0.55	0.5	−1.21	2.21	0.28
BMI		23.72 ± 2.16	23.54 ± 2.00	0.19	0.85	0.18	−1.78	2.14	0.09
Boxing (competitive) experience in years		3.92 ± 0.83	4.35 ± 0.71	1.25	0.213	0.43	−0.3	1.16	0.56
Weekly training sessions		6.4 ± 0.84	6.5 ± 0.74	0.36	0.72	0.1	−0.47	0.67	0.12
1RM Elbow Flexion (Kg)	Dominant	16.70 ± 1.51	17.15 ± 1.70	−0.63	0.54	−0.45	−1.96	1.06	−0.28
	Non-dominant	14.50 ± 1.00	14.70 ± 1.16	−0.41	0.68	−0.2	−1.22	0.82	−0.19
1RM Elbow Extension (Kg)	Dominant	17.40 ± 1.71	17.15 ± 1.92	0.31	0.76	0.25	−1.46	1.96	0.14
	Non-dominant	13.80 ± 0.98	15.10 ± 1.66	−2.13	0.05	−1.3	−2.58	−0.02	−0.95
1RM Back squat (Kg)		82.19 ± 8.22	84.84 ± 8.59	0.71	0.49	−2.65	−10.55	5.25	0.32
1RM Bench press (Kg)		77.19 ± 7.49	79.60 ± 8.30	−0.68	0.50	−2.41	−9.84	5.02	0.31
Peak Punch Force -Jab (N)	Dominant	2638.07 ± 98.74	2635.94 ± 179.93	0.03	0.97	2.13	−134.23	138.49	0.02
	Non-Dominant	2310.16 ± 147.74	2295.11 ± 181.63	0.2	0.84	15.05	−140.49	170.6	0.09
Peak Punch Force - Upper Cut (N)	Dominant	2964.17 ± 40.54	2934.00 ± 52.94	1.43	0.17	30.17	−14.13	74.46	0.64
	Non-Dominant	2490.73 ± 253.39	2571.95 ± 213.34	−0.78	0.45	−81.22	−301.29	138.84	−0.35
Peak Punch Force- Hook (N)	Dominant	3342.50 ± 120.06	3475.90 ± 109.22	−4.55	<0.001	−233.4	−341.23	−125.56	−2.03
	Non-Dominant	2631.40 ± 116.44	2677.53 ± 137.58	−0.81	0.43	−46.13	−165.88	73.61	−0.36
Peak Punch Force - Cross (N)	Dominant	2080.53 ± 79.83	2080.90 ± 85.38	−0.01	0.99	−0.37	−78.02	77.29	−0.004
	Non-Dominant	1951.93 ± 69.67	1936.20 ± 118.47	0.36	0.72	15.73	−75.57	107.04	0.16

Kg, kilogram; N, newton; t, statistical value for independent t-test; p, level of significance (<0.05).

The sample included 60% orthodox and 40% southpaw stance boxers, with 85% being right-handed. Participants represented lightweight (*n =* 8), welterweight (*n =* 7), and middleweight (*n =* 5) divisions. On average, they trained 6.5 ± 1.2 sessions per week, with a weekly training volume of approximately 10–14 h. They had 4.35 ± 0.71 years of competitive experience, completed 25.4 ± 6.7 sanctioned bouts, and had a win ratio of 68.3%.

### 3.2 Paired t-test

**FIGURE 2:**
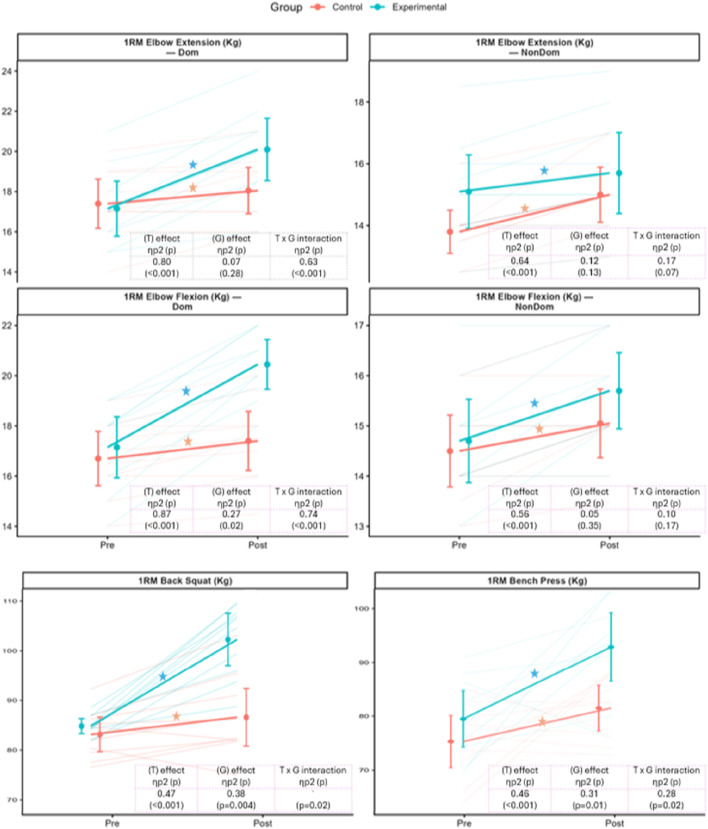
Pre- and post-intervention comparisons of strength outcomes. Orange = Control; Blue = Experimental. Variables include 1RM elbow flexion/extension (dominant and non-dominant), 1RM back squat, and 1RM bench press (kg). Points show group means ± SD with lines linking Pre → Post means; faint thin lines indicate individual trajectories. Side brackets show within-group Pre vs. Post tests (left = Control, right = Experimental; “ns” = p ≥ 0.05; ★, if present, indicates significant change after Bonferroni adjustment). The inset in each panel reports the 2 × 2 repeated-measures ANOVA—Time (T), Group (G), and Time × Group (T × G)—with partial η^2^ and p-values; significant T × G effects are followed by Bonferroni-adjusted *post hoc* contrasts. Overall, the experimental group showed greater improvements across outcomes after the 8-week intervention.

**FIGURE 3:**
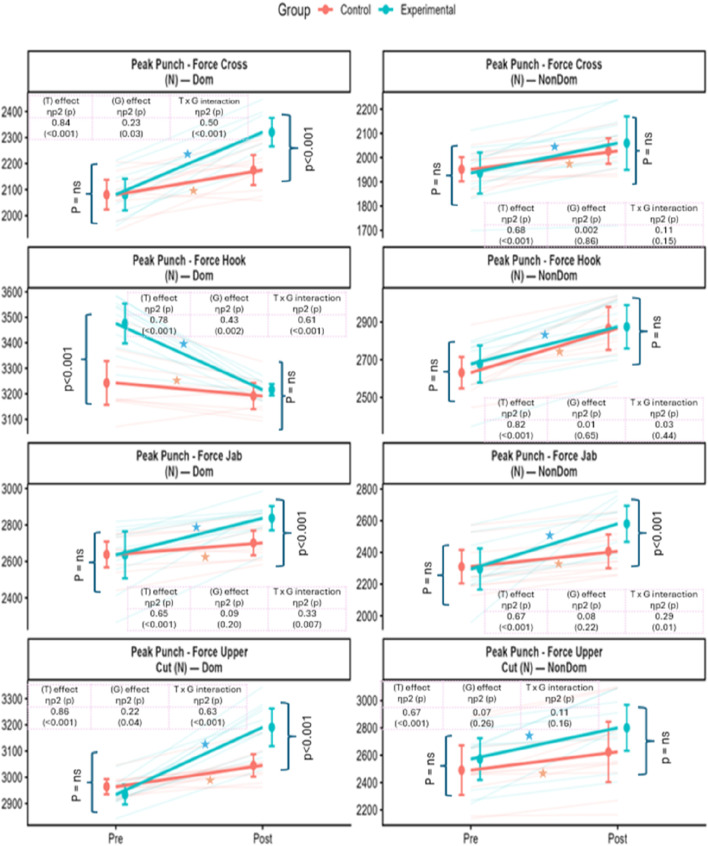
Pre- and post-intervention comparisons of punching-force outcomes. Orange = Control; Blue = Experimental. Variables include peak punch force for jab, cross, hook, and upper-cut, measured in both dominant and non-dominant hands (units: N). Data points show group means ± SD, with lines connecting Pre → Post means; faint thin lines represent individual participant trajectories. Side brackets denote within-group Pre vs. Post comparisons (left = Control, right = Experimental; “ns” = p ≥ 0.05; ★ indicates significant change after Bonferroni adjustment). Insets summarize the results of the 2 × 2 repeated-measures ANOVA, showing main effects of Time (T), Group (G), and the Time × Group interaction (T × G), with partial η^2^ and p-values. Where T × G effects were significant, Bonferroni-adjusted *post hoc* contrasts were conducted. Overall, higher values reflect greater punching force, with the experimental group demonstrating larger gains from Pre to Post.

Both the experimental and control groups demonstrated significant pre-to-post improvements in strength and punching force (see Table 2). However, the experimental group exhibited consistently larger gains across most outcome measures. For upper limb strength, 1RM elbow flexion in the dominant hand increased by 3.3 kg in the experimental group (p < 0.001) compared to 0.7 kg in the control group (p = 0.003). Similarly, elbow extension improved by 2.95 kg in the experimental group (p < 0.001), while the control group showed a smaller gain of 0.65 kg (p = 0.033). Substantial strength gains were also observed in compound lifts, with the experimental group improving their 1RM back squat and bench press by 15.56 kg and 13.59 kg, respectively (both p < 0.001), compared to more modest increases in the control group (see Figure 2). In terms of punching force, the experimental group showed marked improvements across all techniques (see Figure 3). Dominant-hand peak punch force increased by 201.1 N (p = 0.001) for the jab (p = 0.001), 256.7 N (p < 0.001) for the uppercut, and 239.8 N (p < 0.001) for the cross. The control group also demonstrated statistically significant gains, but of smaller magnitude—for example, a 63.3 N increase in jab force (p = 0.006).

**TABLE 2:** Paired t-test for within-group changes in performance, strength, and punching forces.

Measurement	Hand	Group	Pre (Mean ± SD)	Post (Mean ± SD)	Mean diff	95% CI lower	95% CI upper	t	p	d
1RM Elbow Flexion (kg)	Dominant	Control	16.7 ± 1.51	17.4 ± 1.65	−0.7	−1.08	−0.31	−4.12	0.003	1.3
		Experimental	17.15 ± 1.7	20.45 ± 1.38	−3.3	−4.04	−2.56	−10.10	<0.001	3.2
	Non-Dominant	Control	14.5 ± 1.00	15.05 ± 0.96	−0.55	−0.94	−0.16	−3.16	0.012	1.0
		Experimental	14.7 ± 1.16	15.7 ± 1.06	−1	−1.58	−0.42	−3.87	0.004	1.23
1RM Elbow Extension (kg)	Dominant	Control	17.4 ± 1.71	18.05 ± 1.61	−0.65	−1.24	−0.06	−2.51	0.033	0.79
		Experimental	17.15 ± 1.92	20.1 ± 2.17	−2.95	−3.69	−2.21	−8.97	<0.001	2.84
	Non-Dominant	Control	13.8 ± 0.98	15 ± 1.25	−1.2	−1.76	−0.64	−4.81	0.001	1.52
		Experimental	15.1 ± 1.66	15.7 ± 1.83	−0.6	−1.04	−0.16	−3.09	0.013	0.98
1RM Back squat (Kg)		Control	82.19 ± 8.22	85.47 ± 8.36	−3.29	−11.39	4.81	−0.92	0.38	0.40
		Experimental	84.84 ± 8.59	100.40 ± 6.41	−15.56	−22.59	−8.54	−5.01	<0.001	2.05
1RM Bench press (Kg)		Control	77.19 ± 7.49	79.82 ± 8.40	−2.63	−10.33	5.07	−0.77	0.46	0.33
		Experimental	79.60 ± 8.30	93.19 ± 6.58	−13.59	−19.03	−8.15	−5.65	<0.001	1.81
Peak Punch - Force Jab (N)	Dominant	Control	2638.07 ± 98.74	2701.37 ± 94.61	−63.3	−103.87	−22.73	−3.53	0.006	1.12
		Experimental	2635.94 ± 179.93	2837.03 ± 92.9	−201.1	−295.82	−106.38	−4.80	0.001	1.52
	Non-Dominant	Control	2310.16 ± 147.74	2406.83 ± 148.28	−96.67	−127.27	−66.07	−7.15	<0.001	2.26
		Experimental	2295.11 ± 181.63	2580.48 ± 159.68	−285.37	−438.97	−131.77	−4.20	0.002	1.33
Peak Punch Force - Upper Cut (N)	Dominant	Control	2964.17 ± 40.54	3045.24 ± 59.86	−81.07	−120.24	−41.90	−4.68	0.001	1.48
		Experimental	2934 ± 52.93	3190.67 ± 100.39	−256.67	−317.38	−195.96	−9.56	<0.001	3.02
	Non-Dominant	Control	2490.73 ± 253.39	2624.42 ± 308.64	−133.7	−206.11	−61.29	−4.18	0.002	1.32
		Experimental	2571.95 ± 213.34	2800.55 ± 234.5	−228.6	−355.47	−101.73	−4.08	0.003	1.29
Peak Punch Force - Hook (N)	Dominant	Control	3242.5 ± 120.06	3191.17 ± 72.04	51.34	0.07	102.6	2.27	0.05	0.72
		Experimental	3475.9 ± 109.22	3216.23 ± 31.43	259.67	186.76	332.58	8.06	<0.001	2.55
	Non-Dominant	Control	2631.4 ± 116.44	2866.2 ± 159.79	−234.8	−326.08	−143.52	−5.82	<0.001	1.84
		Experimental	2677.53 ± 137.58	2874.93 ± 160.9	−197.4	−254.45	−140.35	−7.83	<0.001	2.48
Peak Punch Force - Cross (N)	Dominant	Control	2080.53 ± 79.83	2175.13 ± 80.39	−94.6	−156.88	−32.32	−3.44	0.007	1.09
		Experimental	2080.9 ± 85.38	2320.7 ± 76.27	−239.8	−284.79	−194.81	−12.06	<0.001	3.8
	Non-Dominant	Control	1951.93 ± 69.67	2027.13 ± 73.52	−75.2	−121.4	−29.00	−3.68	0.005	1.16
		Experimental	1936.2 ± 118.47	2059.8 ± 154.34	−123.6	−178.91	−68.29	−5.06	0.001	1.6

Kg, kilogram; N, newtons; t, statistical value for t-test; p, value of significance (<0.05); d, effect size.

### 3.3 Repeated measures ANOVA

Repeated measures ANOVA revealed significant time effects and group × time interactions across strength and punching force outcomes, indicating differential improvements over the 8-week intervention (see Table 3). For 1RM elbow flexion and extension, both groups improved over time, with significant time effects observed for the dominant arm (Flexion: η^2^ = 0.87, p < 0.001; Extension: η^2^ = 0.80, p < 0.001). Notably, the experimental group showed significantly greater gains in elbow flexion, as reflected in both a group effect (η^2^ = 0.27, p = 0.02) and a strong interaction effect (η^2^ = 0.74, p < 0.001). A similar pattern was seen for elbow extension, where the interaction effect was also substantial (η^2^ = 0.63, p < 0.001), indicating superior adaptations in the BFRT group.

**TABLE 3:** Repeated measures ANOVA for within-group and between group comparisons of pre- and post-performance, strength and punching force.

Measurement	Hand	Group	Pre (Mean ± SD)	Post (Mean ± SD)	(T) effect ηp2 (p)	(G) effect ηp2 (p)	T × G interaction ηp2 (p)
1RM Elbow Flexion (Kg)	Dominant	Control	16.7 ± 1.51	17.4 ± 1.65	0.87 (<0.001)	0.27 (0.02)	0.74 (<0.001)
		Experimental	17.15 ± 1.7	20.45 ± 1.38			
	Non-Dominant	Control	14.5 ± 1.00	15.05 ± 0.96	0.56 (<0.001)	0.05 (0.35)	0.10 (0.17)
		Experimental	14.7 ± 1.16	15.7 ± 1.06			
1RM Elbow Extension (Kg)	Dominant	Control	17.4 ± 1.71	18.05 ± 1.61	0.80 (<0.001)	0.07 (0.28)	0.63 (<0.001)
		Experimental	17.15 ± 1.92	20.1 ± 2.17			
	Non-Dominant	Control	13.8 ± 0.98	15 ± 1.25	0.64 (<0.001)	0.12 (0.13)	0.17 (0.07)
		Experimental	15.1 ± 1.66	15.7 ± 1.83			
1RM Back Squat (Kg)		Control	82.19 ± 8.22	85.47 ± 8.36	0.47 (<0.001)	0.38 (p = 0.004)	0.27 (p = 0.02)
		Experimental	84.84 ± 8.59	100.40 ± 6.41			
1RM Bench Press (Kg)		Control	77.19 ± 7.49	79.82 ± 8.40	0.46 (<0.001)	0.31 (p = 0.01)	0.28 (p = 0.02)
		Experimental	79.60 ± 8.30	93.19 ± 6.58			
Peak Punch - Force Jab (N)	Dominant	Control	2638.07 ± 98.74	2701.37 ± 94.61	0.65 (<0.001)	0.09 (0.20)	0.33 (0.007)
		Experimental	2635.94 ± 179.93	2837.03 ± 92.9			
	Non-Dominant	Control	2310.16 ± 147.74	2406.83 ± 148.28	0.67 (<0.001)	0.08 (0.22)	0.29 (0.01)
		Experimental	2295.11 ± 181.63	2580.48 ± 159.68			
Peak Punch Force - Upper Cut (N)	Dominant	Control	2964.17 ± 40.54	3045.24 ± 59.86	0.86 (<0.001)	0.22 (0.04)	0.63 (<0.001)
		Experimental	2934 ± 52.93	3190.67 ± 100.39			
	Non-Dominant	Control	2490.73 ± 253.39	2624.42 ± 308.64	0.67 (<0.001)	0.07 (0.26)	0.11 (0.16)
		Experimental	2571.95 ± 213.34	2800.55 ± 234.5			
Peak Punch Force - Hook (N)	Dominant	Control	3242.5 ± 120.06	3191.17 ± 72.04	0.78 (<0.001)	0.43 (0.002)	0.61 (<0.001)
		Experimental	3475.9 ± 109.22	3216.23 ± 31.43			
	Non-Dominant	Control	2631.4 ± 116.44	2866.2 ± 159.79	0.82 (<0.001)	0.01 (0.65)	0.03 (0.44)
		Experimental	2677.53 ± 137.58	2874.93 ± 160.9			
Peak Punch Force - Cross (N)	Dominant	Control	2080.53 ± 79.83	2175.13 ± 80.39	0.84 (<0.001)	0.23 (0.03)	0.50 (<0.001)
		Experimental	2080.9 ± 85.38	2320.7 ± 76.27			
	Non-Dominant	Control	1951.93 ± 69.67	2027.13 ± 73.52	0.68 (<0.001)	0.002 (0.86)	0.11 (0.15)
		Experimental	1936.2 ± 118.47	2059.8 ± 154.34			

Kg, kilogram; N, newton; p, level of significance (<0.05); T, time; G, group.

Strength gains in compound lifts were also more pronounced in the experimental group (see Figure 2). The 1RM back squat and bench press demonstrated significant group effects (Squat: η^2^ = 0.38, p = 0.004; Bench press: η^2^ = 0.31, p = 0.01) and interaction effects (Squat: η^2^ = 0.27, p = 0.02; Bench press: η^2^ = 0.28, p = 0.02), confirming greater strength improvements over time relative to the control group.

For Peak Punching force (N), significant time effects were observed for all punch types, particularly in the dominant hand (see Figure 3). The most substantial improvements were seen for the uppercut (η^2^ = 0.86, p < 0.001) and cross (η^2^ = 0.84, p < 0.001). Interaction effects were most prominent for the jab (η^2^ = 0.33, p = 0.007) and uppercut (η^2^ = 0.63, p < 0.001), suggesting that the BFRT protocol had a differential impact on technique-specific punching force development. While group effects were less consistent for punch force, the timing and magnitude of improvements favored the experimental group.

**FIGURE 4:**
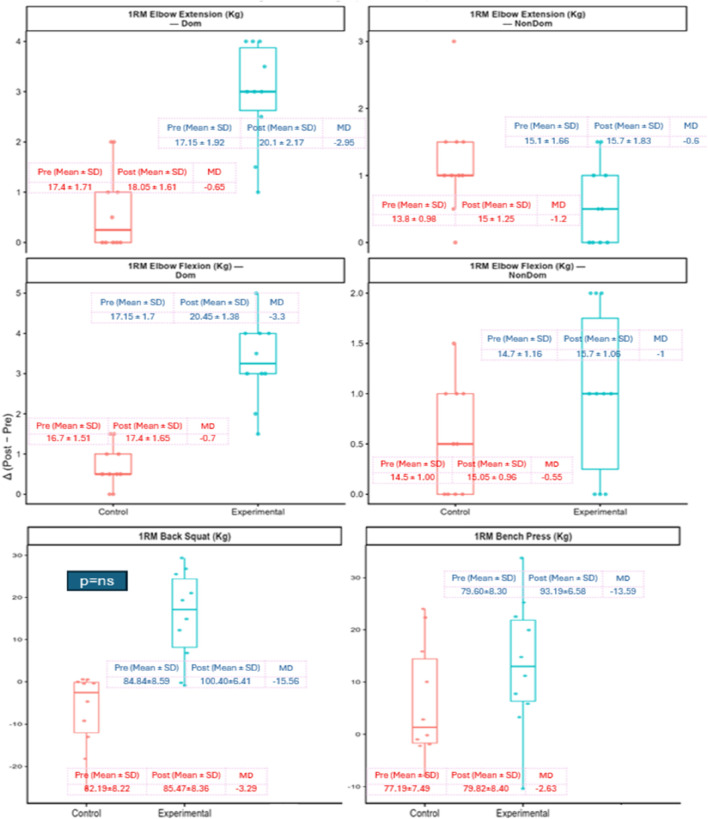
Change in strength outcomes from pre- to post-intervention (Δ = Post − Pre). Orange = Control; Blue = Experimental. Variables include one-repetition maximum (1RM) elbow extension and flexion (dominant and non-dominant), 1RM back squat, and 1RM bench press (kg). Boxplots display the distribution of individual change scores, with boxes representing interquartile ranges, horizontal lines the median, and whiskers the range; individual participant data points are overlaid. Insets show group means ± SD at Pre and Post, together with mean differences (MD). Overall, the experimental group demonstrated larger gains across most strength measures compared with control, though some improvements were modest or non-significant.

**FIGURE 5:**
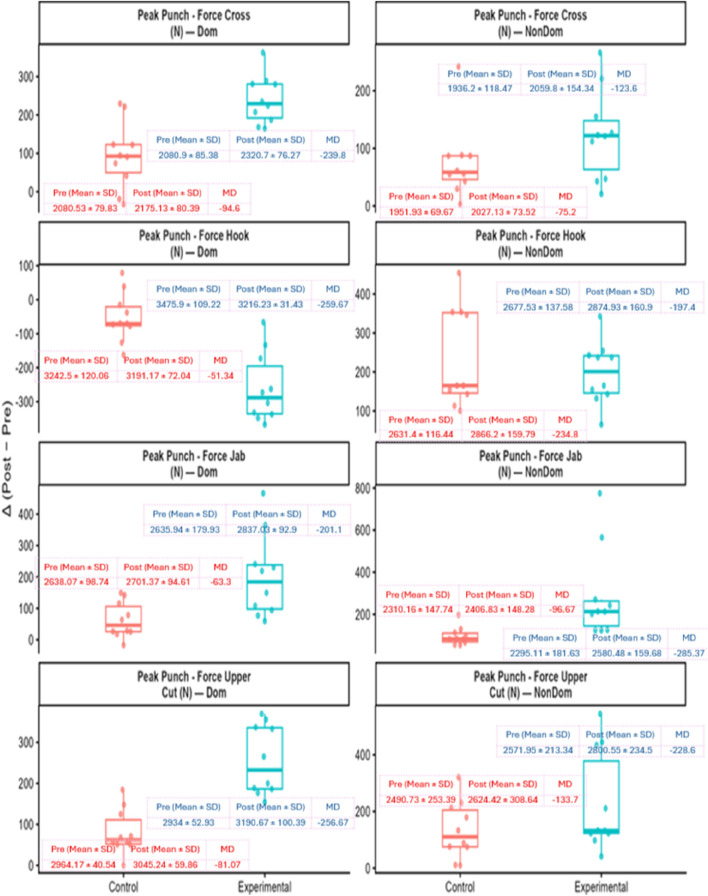
Change in peak punching force from pre- to post-intervention (Δ = Post − Pre). Orange = Control; Blue = Experimental. Variables include cross, hook, jab, and uppercut for dominant and non-dominant hands (N). Boxplots show interquartile range, median, whiskers, and individual data. Insets display group means ± SD at Pre and Post with corresponding mean differences. Overall, the experimental group exhibits larger positive Δ for most punches, while dominant-hand Hook decreases in both groups.

Change-score plots provided further support for these findings. The experimental group demonstrated consistently greater Δ (Post−Pre) across strength outcomes, with the largest gains evident in compound lifts compared with the control group (see Figure 4). Similarly, larger positive Δ in punching force were observed for jabs, crosses, and uppercuts in the experimental group, while the dominant-hand hook declined in both groups (see Figure 5).

## 4 Discussion

This randomized controlled trial demonstrated that Blood Flow Restriction Training (BFRT) produced significantly greater improvements in upper limb 1RM strength (elbow flexion, extension, bench press) and peak punch-specific force (jab, cross, hook, uppercut) compared to a moderate-load, high-repetition control protocol (50%–60% 1RM). The control program was therefore more aligned with strength endurance training, which may partly explain the smaller magnitude of gains observed in that group. Nonetheless, BFRT produced meaningful improvements in maximal strength and punch-specific force, consistent with evidence that low-load BFRT (20%–30% 1RM at 40%–50% LOP) can elicit adaptations comparable to traditional high-load resistance training. These results support BFRT as a safe and efficient low-load method to enhance boxing performance, particularly in phases where joint-sparing strategies are desirable, as adaptations can be achieved with reduced mechanical load (Hughes et al., 2017; Loenneke et al., 2011).

Boxing requires a combination of explosive power, technical precision, and neuromuscular coordination to deliver impactful punches. Strength development, particularly in the upper body, plays a vital role in optimizing punch velocity and force transmission through the kinetic chain—from the lower limbs through the core to the upper extremities (Lenetsky et al., 2020; Stanley, 2020). Traditional high-load resistance training has been widely used to develop these qualities; however, it carries a risk of joint overload and fatigue accumulation, especially during intense competition phases. The present study offers evidence that low-load BFRT can provide comparable or superior strength gains with reduced external load, making it a viable alternative for high-performance athletes.

Significant improvements were observed in both proximal and distal strength outcomes in the BFRT group, including 1RM elbow flexion, elbow extension, bench press, and back squat. These gains were evident in both dominant and non-dominant limbs, suggesting bilateral adaptation. Such results align with previous findings indicating that BFRT can stimulate hypertrophy and strength development comparable to traditional high-load protocols due to its distinct physiological mechanisms, including metabolic stress and hypoxic muscle stimulation (Chang et al., 2023; Pavlou et al., 2023). The capacity to induce meaningful adaptations at 20%–30% of 1RM is particularly relevant in boxing, where cumulative impact stress can limit high-intensity strength training volume. This is supported by evidence showing that low-load BFRT at 20%–30% of 1RM can elicit significant improvements in maximal strength and power, reinforcing the appropriateness of the current protocol for evaluating 1RM and sport-specific outcomes (Pavlou et al., 2023; Pearson and Hussain, 2015; Yasuda et al., 2011).

The selection of upper body resistance exercises in this study—bench press, front raises, flys, and triceps extensions—targeted key muscles involved in punch execution. For instance, the bench press recruits the pectoralis major, deltoid, and triceps brachii to support horizontal force output during jabs and crosses. In contrast, front raises emphasize anterior deltoid activation, which is essential for initiating hooks and uppercuts (Stanley, 2020). The substantial post-intervention improvements in these strength measures in the BFRT group further highlight its effectiveness in targeting boxing-relevant muscle groups.

The observed performance enhancements may be attributed to the unique adaptations induced by BFRT. The technique increases intramuscular metabolic stress through occlusion, promoting the release of growth hormone, recruitment of fast-twitch motor units, and muscle protein synthesis (Jessee et al., 2018; Pearson and Hussain, 2015; Schoenfeld et al., 2023; Scott et al., 2015). BFRT has also been associated with a shift toward type IIb muscle fibers, which are critical for explosive movements such as punching (Saraf et al., 2022). These adaptations may explain the substantial increases in punch force observed across all punch types—jab, cross, hook, and uppercut—in both dominant and non-dominant arms in the BFRT group.

This study is among the few to directly evaluate punch-specific force outcomes using a calibrated Vernier force plate embedded in a suspended heavy bag, offering field-based relevance. While this sport-specific setup enhances ecological validity, it may underestimate absolute force values compared with rigid wall-mounted platforms such as Bertec systems (Finlay et al., 2023) or Loadstar Sensors devices (Omcirk et al., 2023). Nevertheless, consistent use of the same apparatus across all participants ensured reliability for within-subject comparisons. The BFRT group outperformed the control group across all punch types, reinforcing the importance of strength training for improving sport-specific force output (Chaabène et al., 2015; Loturco et al., 2016).

Although the control group demonstrated modest improvements in some strength and punch force measures, these changes were significantly smaller in magnitude and lacked consistent interaction effects. This suggests that traditional resistance training at moderate intensities may be less effective than BFRT in eliciting rapid adaptations in elite boxing populations.

This study has several limitations that may affect the generalizability of its findings. Although an *a priori* power analysis was conducted, the final sample size (20 participants, 10 per group) fell slightly short of the 24 required by the *a priori* power analysis, due to 8 withdrawals during the intervention. While this may have reduced statistical power, the study remained adequately powered to detect moderate effects. Participants were exclusively male boxers from a single academy, introducing potential sampling bias. This restricts external validity in three ways: the absence of female athletes limits insights into sex-specific adaptations; results may differ across competition levels (from novice to professional) due to varied training histories; and applicability to international contexts is uncertain, given differences in coaching systems and cultural environments. The short intervention duration limited the assessment of long-term retention. Blood pressure was monitored during BFRT sessions to ensure participant safety; however, structured cardiovascular outcomes such as heart rate variability or exertional indices were not formally analyzed. In addition, the study did not include direction measures of fatigue resistance–a key performance quality in boxing or systematic assessments of perceptual fatigue, which may have provided further insight into endurance-related adaptations. Punching force was measured using a Vernier force plate mounted in a suspended heavy bag, which may underestimate absolute values compared to rigid wall-mounted platforms; however, consistency across participants ensured reliable within-subject comparisons. These limitations highlight the need for future multi-center trials with larger, more diverse cohorts and broader outcome measures.

## 5 Conclusion

This study demonstrates that Blood Flow Restriction Training (BFRT) can significantly enhance punching force and upper extremity strength in elite male boxers. Over the 8-week training period, boxers in the BFRT group exhibited superior improvements in key performance metrics compared to the control group undergoing traditional resistance training. These enhancements were evident in increased muscle strength across the upper limbs, reinforcing BFRT’s effectiveness in improving both localized and total punching power. Importantly, the results align with previous evidence showing that low-load BFRT protocols at ∼20–30% of 1RM are capable of eliciting maximal strength and power adaptations, thereby supporting the validity of the chosen intervention. Given its ability to stimulate muscular adaptations at low intensities with minimal joint stress, BFRT presents a practical and safe strategy for performance enhancement in elite boxing.

From a practical standpoint, coaches can consider integrating BFRT into boxing conditioning cycles as a tool for tapering and load management. During taper phases, BFRT at 20%–30% 1RM may help preserve or even enhance strength and punching force while reducing mechanical stress and fatigue accumulation before competition. In high-volume training blocks, it can serve as a joint-sparing strategy, offering meaningful adaptations at lower external loads. Practically, two to three supervised sessions per week, lasting 30–40 min and focused on boxing-relevant resistance exercises (bench press, triceps extension, front raises, etc.), can be incorporated without disrupting technical or tactical work.

Future research should explore the long-term efficacy and retention of BFRT-induced adaptations through extended follow-up protocols. Studies involving larger, more diverse cohorts—including female athletes and varying age or competition levels—are essential to improve external validity. Comparative trials evaluating BFRT against other resistance and conditioning approaches will help optimize evidence-based programming. Integrating biomechanical analysis, cardiovascular assessments, and psychological profiling could offer a holistic understanding of BFRT’s impact. Further investigation into the optimal parameters of BFRT—such as cuff width, pressure, and exercise selection—will refine its application across various settings, including athletic performance, rehabilitation, and sports medicine.

## Data Availability

The original contributions presented in the study are included in the article/supplementary material, further inquiries can be directed to the corresponding author.
